# Domains I and IV of Annexin A2 Affect the Formation and Integrity of *In Vitro* Capillary-Like Networks

**DOI:** 10.1371/journal.pone.0060281

**Published:** 2013-03-29

**Authors:** Aase M. Raddum, Lasse Evensen, Hanne Hollås, Ann Kari Grindheim, James B. Lorens, Anni Vedeler

**Affiliations:** 1 Department of Biomedicine, University of Bergen, Bergen, Norway; 2 Centre for Pharmacy, University of Bergen, Bergen, Norway; 3 Molecular Imaging Center (MIC), University of Bergen, Bergen, Norway; INRS, Canada

## Abstract

Annexin A2 (AnxA2) is a widely expressed multifunctional protein found in different cellular compartments. In spite of lacking a hydrophobic signal peptide, AnxA2 is found at the cell surface of endothelial cells, indicative of a role in angiogenesis. Increased extracellular levels of AnxA2 in tumours correlate with neoangiogenesis, metastasis and poor prognosis. We hypothesised that extracellular AnxA2 may contribute to angiogenesis by affecting endothelial cell-cell interactions and motility. To address this question, we studied the effect of heterotetrameric and monomeric forms of AnxA2, as well as its two soluble domains on the formation and maintenance of capillary-like structures by using an *in vitro* co-culture system consisting of endothelial and smooth muscle cells. In particular, addition of purified domains I and IV of AnxA2 potently inhibited the vascular endothelial growth factor (VEGF)-dependent formation of the capillary-like networks in a dose-dependent manner. In addition, these AnxA2 domains disrupted endothelial cell-cell contacts in preformed capillary-like networks, resulting in the internalisation of vascular endothelial (VE)-cadherin and the formation of VE-cadherin-containing filopodia-like structures between the endothelial cells, suggesting increased cell motility. Addition of monoclonal AnxA2 antibodies, in particular against Tyr23 phosphorylated AnxA2, also strongly inhibited network formation in the co-culture system. These results suggest that extracellular AnxA2, most likely in its Tyr phosphorylated form, plays a pivotal role in angiogenesis. The exogenously added AnxA2 domains most likely mediate their effects by competing with endogenous AnxA2 for extracellular factors necessary for the initiation and maintenance of angiogenesis, such as those involved in the formation/integrity of cell-cell contacts.

## Introduction

Annexin A2 (AnxA2) is a conserved 36 kDa multifunctional protein [1], [2], [3], [4]. The core structure of AnxA2 consists of four tightly packed domains, each comprising five α-helices [5]. The protein also contains a unique N-terminal domain of about 30 amino acids, which forms a structurally separate unit. AnxA2 is localised to different cellular compartments [1]. In the cytoplasm, it may associate with cytoskeletal actin filaments. In addition, it is found at the plasma membrane and localises to compartments of the biosynthetic and endocytic pathways, indicating that it is involved in membrane traffic or other membrane events [1], [2], [3], [6]. Localisation of AnxA2 to the extracellular surface of the plasma membrane, facilitated by lipid raft endocytosis and exosomal transport [7], has been found in many cell types, including endothelial cells (ECs) [8], macrophages [9] as well as cancer cells associated with lymphoma [10] or leukemia [11]. AnxA2 exerts its multiple functions through binding to different intra- and extracellular ligands. These functions are modulated via numerous post-translational modifications, including Tyr and Ser phosphorylation [1], [2], [3], indicating that the protein plays an important role in signal transduction. The ligands include Ca^2+^ [1], [2], [3], anionic phospholipids [12], cholesterol [13], S100A10 (p11) [14], tissue plasminogen activator (tPA) and plasminogen/plasmin [15], F- and G-actin [16], [17], vascular endothelial (VE)-cadherin [18], specific mRNAs [19] and heparin [20]. The large number of ligands and posttranslational modifications of AnxA2 reflect its multicompartmental nature and multifunctionality.

AnxA2 and its main ligand, S100A10, form a heterotetramer. In ECs and epithelial cells at least 90% of the total cellular AnxA2 is present in such a heterotetrameric form [21]. The AnxA2-S100A10 heterotetramer can connect two membrane surfaces, as it harbours two annexin phospholipid-binding cores that are bridged by an S100A10 dimer [2], [8]. AnxA2 has been implicated in the organisation and dynamics of lipid rafts, as it binds, perhaps indirectly, cholesterol in rafts at sites of actin recruitment [13], [22], [23]. Thus, it may act as a scaffolding linker protein that organises the interface between the cytoskeleton and the cytoplasmic face of cellular membranes [6], [24], [25]. AnxA2 interacts with VE-cadherin [18], which is an EC-specific cadherin that constitutes the main component of inter-endothelial adherens junctions. VE-cadherin is involved in the control of vascular permeability and may associate with the VEGF receptor 2, most likely as a consequence of its Tyr phosphorylation [26]. As an important part of the maintenance of VE-cadherin at the inter-endothelial adherens junctions, AnxA2 binds to both the actin cytoskeleton and the VE-cadherin-based complex, thereby locking the latter to the cholesterol-containing rafts [18].

Angiogenesis is the process that generates new blood vessels through sprouting of ECs from pre-existing vessels. It plays an important role during wound healing and is also an underlying process in a number of pathological states such as cancer, diabetic retinopathy and psoriasis [27]. It has been suggested that extracellular AnxA2 plays a role in angiogenesis, by acting as a cell surface co-receptor for plasminogen and tPA, thereby contributing to local plasmin generation [15]. In addition to matrix metalloproteinases, the production of plasmin and other proteases of the plasminogen activator system is required for the localised breakdown of the extracellular matrix (ECM) during the sprouting of new blood vessels [28]. Anti-angiogenic therapeutics targeting VEGF are used clinically to treat several forms of cancer.

We hypothesised that exogenously added soluble AnxA2 could act as a dominant negative competitor of extracellular AnxA2 by competing for ligands and thus block the ensuing cellular response. To address this possibility, we employed a co-culture system of ECs and smooth muscle cells (SMCs) that mimics several of the VEGF-dependent EC functions during the initial phases of angiogenesis, including cell migration and morphogenesis [29]. The resulting capillary-like endothelial network is joined via VE-cadherin adherens junctions and surrounded by vascular basement membrane proteins. This deposition of a complex basement membrane leads to an endothelial phenotype that is largely VEGF-independent [29], allowing the assessment of both VEGF-dependent (in the initial phases) and VEGF-independent (after 72 h of co-culture) cell functions. In fact, this differential VEGF-dependency of the forming and established blood vessels could explain their diminishing responsiveness to the inhibitors of VEGF signalling that are in clinical use [30], [31]. The *in vitro* networks can be visualised by live cell imaging using automated microscopy systems and network parameters can be quantified by image analysis [32], [33]. This provides an ideal system for the initial screening of anti-angiogenic agents [33].

Recent studies have raised the concern that depriving tumours of oxygen by inhibiting angiogenesis may lead to subsequent tumour growth and metastasis [34]. Hypoxia-inducible factor-1α (HIF-1α), a master transcriptional regulator of oxygen-sensitive genes, is overexpressed in hypoxic tumours [34] and increases the transcription of several genes, including VEGF [35] and *anx*A2 [36]. The VEGF-targeted therapies to inhibit angiogenesis may induce severe side effects and lead to more invasive and metastatic tumours [37] explaining their unsuccessfulness so far. Since AnxA2 is involved in both angiogenesis and metastasis [38], [39], [40], it may serve as a potential target for the simultaneous inhibition of both processes.

## Materials and Methods

### Expression and purification of AnxA2, S100A10 and domains I and IV of AnxA2

The bovine *anx*A2 cDNA was used as a template for the cloning and expression of wt HisAnxA2 and wt HisMBP-AnxA2-D_I_ in the pETM (inserted in the *Nco*I and *Acc65*I sites) and pETM-41 vector, respectively. All constructs were verified by DNA sequencing. The pETM vectors were kindly provided by Prof. Gunter Stier (University of Heidelberg, Germany). The pET28a vector containing human His-tagged S100A10 wt cDNA was kindly provided by Prof. Volker Gerke (University of Münster, Germany). The methods for expression and purification of HisMBP-AnxA2-D_I_ and HisMBP-AnxA2-D_IV_ fusion proteins have been explained in detail [41]. Cleaved AnxA2-D_I_ (GAM^25^GSVKAYTNFDAERDALNIETAIKTKGVDEVTIVNILTNRSNEQRQDIAFAYQRRTKKELASALKSALSGHLETVILGLLKT) and the soluble mutant AnxA2-D_IV_ (GAM^264^QNKPLSYADRLYDSMKGKGTRDKVLIRIMVSRSESDMSKIRSEFKKKYGKSLYYYIQQDTKGDYQKALLSLCGGDD; the mutated amino acid residues are underlined) [41] were purified and separated from HisMBP and the uncut fusion protein by using Q-Sepharose (Amersham Biosciences).

### Purification of the AnxA2 heterotetramer

The AnxA2-S100A10 heterotetramer was purified from epithelial cells derived from pig intestines as described [42].

### EGTA-mediated release of AnxA2 from the extracellular surface of the plasma membrane

Human umbilical vein endothelial cells (HUVECs) in monoculture were harvested by centrifugation at 800× g for 5 min. The cells were washed twice in PBS before EGTA-release of proteins in the presence of 200 µM orthovanadate (only control) and a protease inhibitor cocktail (Roche; EDTA-free).

### Actin overlay assays

AnxA2-D_I_, AnxA2-D_IV_, and wt AnxA2 were separated by 15% SDS-PAGE and transferred to a nitrocellulose membrane. Since denatured AnxA2 does not bind actin (results not shown), proteins were partly renatured by the method of Chen et al. [43]. Subsequently, the blot was incubated ON with 10 µg/ml actin (Cytoskeleton Inc.) in TBS/Tween buffer (10 mM Tris-base of pH 8.0, 150 mM NaCl, 0.1% Tween 20) with 3% non-fat dry milk and 1% glycine. After thorough washing, the blot was incubated with monoclonal actin antibodies (clone C4; ICN Biomedicals) and actin binding was detected by using HRP-conjugated anti-mouse secondary antibodies and enhanced chemiluminescence substrate (Pierce, Thermo Scientific).

### Cell culture

Green- (GFP) or Red Fluorescent Protein (RFP)- expressing HUVECs (Lonza; C2517A) and pulmonary artery SMCs (Lonza; CC2581) were maintained in culture in the supplier's recommended complete medium (EGM-2 or SMGM-2; Lonza) at 37°C in 5% CO_2_ atmosphere. The maximum passage number of cells used for experiments was 9 (HUVECs) and 10 (SMCs).

### Co-culture assay

The co-culture assay has been described in detail elsewhere [29]. Briefly, the HUVEC and SMCs were seeded simultaneously, treated and cultured in EGM-2 for at least 72 h to ensure stable network formation. Networks were imaged by fluorescence microscopy after 72 h of treatment.

### Treatment of co-cultures

The S100A10, AnxA2-S100A10 heterotetramer, AnxA2 monomer, AnxA2-D_I_ and AnxA2-D_IV_ were added to the co-cultures 2 h after seeding (or 96 h for preformed networks) at a concentration of 5–15 µM as indicated. Purified monoclonal mouse anti-Annexin II (BD Biosciences), p-Annexin II (85.Tyr 24) (Santa Cruz Biotechnologies) and Annexin II (C-10) (Santa Cruz Biotechnologies) were passed through a Zeba spin desalting column (Thermo Scientific), equilibrated with 20 mM Tris of pH 8, to remove traces of Na-azide. The antibodies were subsequently added to the co-culture medium at a concentration of 20 µg/ml.

### Plasminogen activation assays on fibrin clots in trans-wells

Fibrin polymerisation was initiated by mixing 10 µl fibrinogen (30 mg/ml; bovine plasma, Sigma) in 1 ml serum-free plain culture medium (EBM-2) and subsequent addition of 10 µl thrombin (100 U/ml; bovine plasma, Sigma). The 24-well cell culture inserts (0.3 cm^2^, 8 µm pore size filters, BD Fluoroblok) were coated with 70 µl of the mixture (0.3 mg/ml fibrin) and left to polymerise for 30 min at 37°C. Subsequently, the inserts were rinsed with EBM-2 and further incubated with complete EGM-2 medium (with serum) for 5 min to inactivate thrombin. 5×10^4^ GFP-expressing HUVECs were seeded in diluted growth factor depleted EGM-2 medium, (1/4 complete EGM-2, 3/4 EGM-2 without growth factors), on top of the filters with the fibrin matrix. The lower chambers contained EGM-2 medium with 10 ng/ml VEGF (R & D Systems) as a chemoattractant. HUVECs were incubated for 30 min at 37°C in the presence of AnxA2-D_I_, AnxA2-D_IV_, lysozyme or PTK787, before seeding in the cell culture insert. These substances were added to both the upper and the lower chambers at the concentrations indicated. After 16 h, the migrated cells on the underside of the filter were visualised using a Nikon TE2000 inverted fluorescence microscope.

### Microscopy and image analysis

For quantitative analysis of the co-cultures, a BD Pathway 855 bioimaging system (BD Biosciences, San Jose, Ca) was used allowing automated high throughput imaging. Statistical analysis of the acquired images was carried out using the BD Image Data Explorer software. Images were acquired as 2×2 montages using a 10× lens with excitation filter 448/10 and emission filter 520/35. During image analysis, fluorescent cells were detected by thresholding and rolling ball filtering (rolling ball 25×25). Statistics on tube total length was obtained using the “Tube Formation” image analysis module of AttoVision v1.6.1. The software produces a one-pixel wide representation in the centre of the cellular network of the GFP-expressing ECs (cellular tubes). Thus, the parameter “tube total length” is the total length of the tubes in pixels with a width of 1 pixel [32]. The images from the trans-well experiments were subjected to a cell counting analysis using Image J. All data are representative of at least three independent experiments giving similar results. The results are expressed as the mean (± SEM) from at least three experiments. Statistical significance was determined using the two-tailed Student's t-test and p-values <0.05 were considered as significant.

### Immunofluorescence staining and confocal microscopy

40×10^3^ HUVECs in mono-culture or co-culture (40×10^3^) with 200×10^3^ SMCs were grown on glass coverslips, fixed for 20 min with 3% paraformaldehyde, permeabilised for 5 min in 0.05% Triton X-100 in PBS at RT and nonspecific binding of antibodies was blocked in PBS containing 0.2% bovine serum albumin (fraction V) and 5% goat serum. To expose antigenic sites for the mouse monoclonal anti-Annexin II (BD Biosciences; used at 1∶100 dilution) the permeabilised cells were incubated for 5 min with 6 M guanidine-HCl in 50 mM Tris, pH 7.4. The staining of guanidine-HCl treated cells with rabbit monoclonal VE-cadherin antibody (D87F2 from Cell Signaling used at 1∶400 dilution) was indistinguishable from that obtained with untreated cells (results not shown). After staining with primary antibodies and rinsing, the cells were incubated with fluorochrome-coupled secondary antibodies (FITC-conjugated goat-anti-rabbit and Alexa Fluor 594-conjugated goat-anti-mouse from Jackson ImmunoResearch Laboratories used at 1∶50 dilution). Finally, the coverslips were inverted on objective glasses on a small drop of Vectashield mounting medium with 4′,6-diamino-2′-phenylindole (DAPI) (Vector Laboratories). Confocal imaging was performed using Leica SP5 AOBS confocal laser scanning microscope. Optical sections were obtained using either 40x/1.25 NA, 63x/1.4 NA or 100x/1.4 NA HCX Plan-Apochromat oil-immersion objectives and ∼1 Airy unit pinhole aperture. Fluorescence images were acquired with 405 Diode, Argon and Helium Neon lasers. Intensity quantification was performed in the Quantify mode of the LAS AF Lite software. Intensity line profiles were calculated for the selected regions of interest.

## Results

### Exogenous AnxA2 associated with S100A10 potently inhibits the formation of in vitro capillary-like networks

**Figure 1:**
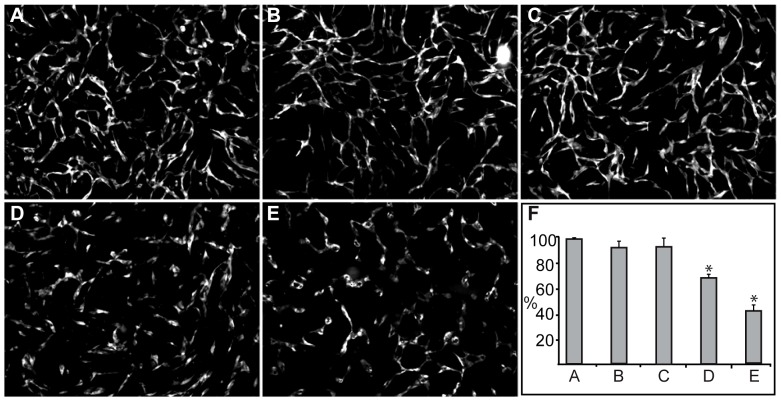
The effects of AnxA2, S100A10 and the heterotetrameric AnxA2_2_-S100A10_2_ complex on the formation of an *in vitro* capillary-like network. Co-cultures of SMCs and GFP-expressing HUVECs were untreated (A), or treated with 15 µM AnxA2 (B), 15 µM S100A10 (C), 6 µM AnxA2_2_-S100A10_2_ complex (12 µM AnxA2) (D) or 100 nM PTK787 (E) at 2 h after seeding. After 72 h incubation, images were taken at 10× magnification. The tube total length was analysed (F) and expressed as percentage relative to the untreated EC control (100%) (A) using the Attovision and BD Image Data Explorer programmes. Results (F) are the mean ± SEM of 3 independent experiments each. Statistical significance was determined by the two-tailed Student's t-test (*P<0.05).

AnxA2, both in its monomeric form and as a heterotetramer with S100A10, has been shown to be involved in angiogenesis [8], [44]. Therefore, we first investigated the effects of the two proteins (AnxA2 and S100A10), either added alone or together in the form of the heterotetrameric complex isolated from porcine intestinal epithelial cells [45], on the formation of a capillary-like network in the co-culture system consisting of ECs and SMCs. As compared to control cells (Figure 1A), AnxA2 (15 µM) and S100A10 (15 µM) (purified form shown in Figure S1) did not inhibit the network formation, while a nearly equimolar concentration of AnxA2 (12 µM), present in the heterotetrameric complex (AnxA2_2_-S100A10_2_) (6 µM), inhibited network formation in the co-culture system by ∼30% (Figure 1, D and F). An equal volume of the buffer (20 mM Tris, pH 8) used to dissolve the proteins was added to the control cultures to rule out buffer effects on network formation. The inhibitory effects of the proteins were also compared to that of PTK787 (ZK222584), a specific inhibitor of the VEGF receptor Tyr kinases [46]. PTK787 has previously been shown to potently inhibit the formation of a capillary-like network in the same co-culture system [29]. In this system, 100 nM PTK787 inhibited network formation by 60–90% (Figure 1, E, F and [29]).

### Domains I and IV of AnxA2 inhibit network formation in a dose-dependent manner

**Figure 2:**
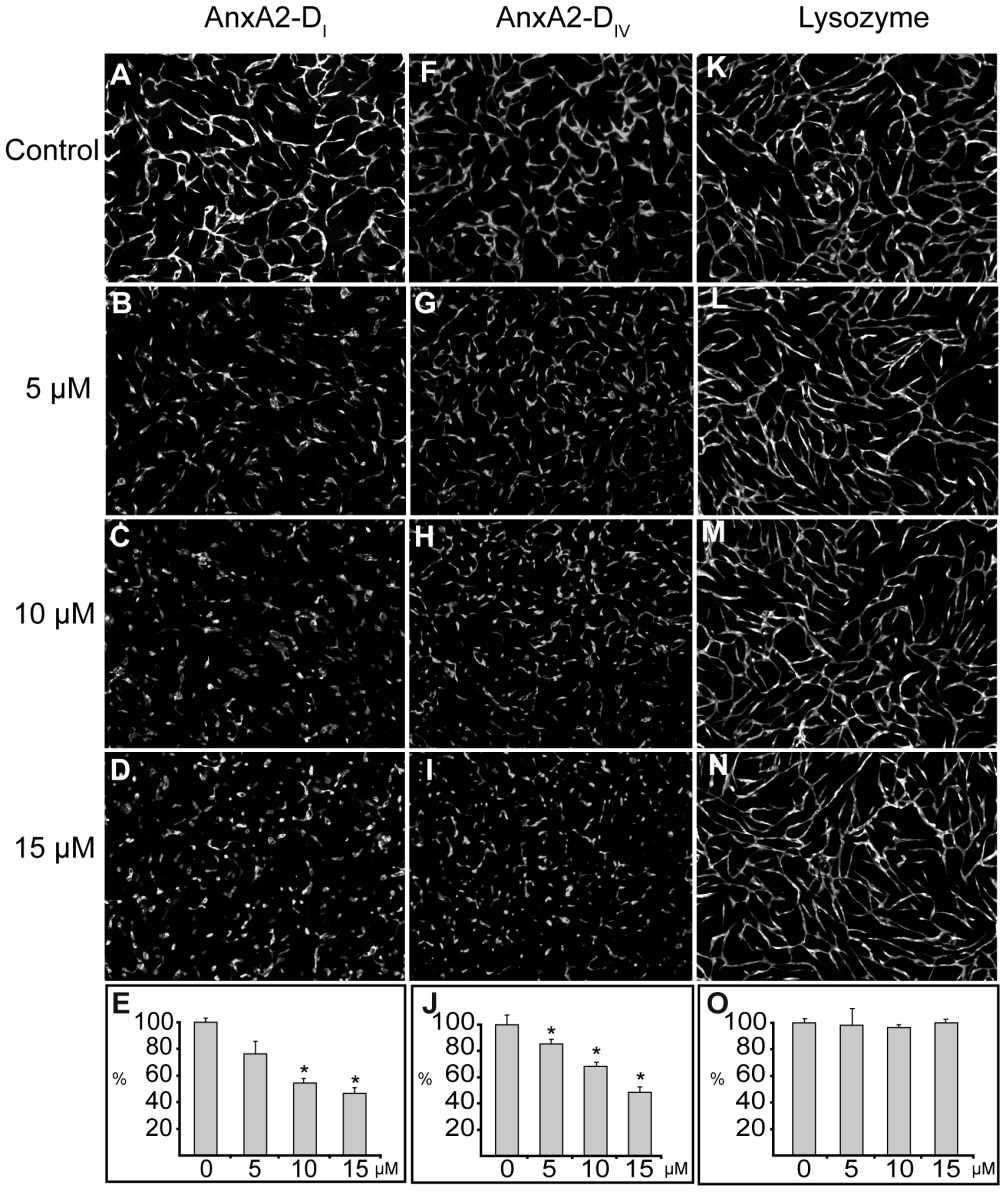
The effects of soluble AnxA2-D_I_, AnxA2-D_IV_ and lysozyme on the formation of an *in vitro* capillary-like network. Co-cultures of SMCs and GFP-expressing HUVECs were treated with 5–15 µM AnxA2-D_I_ (**B**–**D**), AnxA2-D_IV_ (**G**–**I**), lysozyme (**L**–**N**) at 2 h after seeding. Panels **A**, **F** and **K** show the corresponding controls with untreated cells. After 72 h incubation, images were taken at 10× magnification. The tube total length was analysed (**E**, **J** and **O**) and expressed as percentage relative to the corresponding untreated EC controls (100%) (**A**, **F** and **K**, respectively), as described for Figure 1. Results (**E**, **J** and **O**) are the mean ± SEM of 3 independent experiments each. Statistical significance was determined by the two-tailed Student's t-test (*P<0.05).

Each of the domains of AnxA2 (9–10 kDa) are of similar size as S100A10 (11 kDa). The N-terminal tail of AnxA2 is flexible and may mask putative binding sites in the full-length protein, which could be exposed as a consequence of its binding to S100A10, thus explaining the effect of the heterotetrameric complex of AnxA2 and S100A10 (Figure 1D). Domain I of AnxA2 (AnxA2-D_I_) may contain part of the tPA-binding site [47] and can be expressed separately in a soluble and fully folded state as determined by Far-UV circular dichroism (CD) and an co-operative unfolding (T_m_) (Figure S2) and nuclear magnetic resonance (NMR) spectroscopy (results not shown), as previously shown for the corresponding domain I of AnxA1 [48]. Domain IV of AnxA2 (AnxA2-D_IV_) harbours the plasminogen/plasmin binding site [8], and has also been reported to contain the actin-binding site(s) [17], [49]. Taken together, these data suggest that the inhibitory effect of the AnxA2 heterotetrameric complex on network formation in the co-culture system is mediated by the AnxA2 moiety. We have been able to produce a soluble and partially folded AnxA2-D_IV_ by mutating hydrophobic amino acid residues involved in interfacial contacts with the other domains [41]. Thus, to identify the regions of AnxA2 responsible for the anti-angiogenic effect of the exogenously added molecule, we next studied the influence of the soluble and folded AnxA2-D_I_, as well as the soluble and partially folded AnxA2-D_IV_ [41], on network formation in the co-culture system. The domains were added at three different concentrations (5, 10 and 15 µM) to detect possible dose-dependent effects (Figure 2). As shown in Figure 2, AnxA2-D_I_ (B–D) and AnxA2-D_IV_ (G–I) inhibit in a dose-dependent manner the formation of a capillary-like network as compared to the corresponding untreated co-cultures (A and F, respectively). The degree of inhibition exerted by the two AnxA2 domains appears to be of similar magnitude (Figure 2, compare E and J). To exclude the possibility that the effects of the domains were simply due to their size or chemical properties, lysozyme was added to the co-culture system at the same concentrations (5, 10 and 15 µM) (Figure 2, L–N). Domains I and IV of AnxA2 have pIs of 8.9 and 9.3 (calculated using the ExPASy server), respectively, while lysozyme has a pI of 9.4. Thus, like the two AnxA2 domains, lysozyme is a small and basic protein. However, lysozyme has no effect on the co-culture system (Figure 2, L–O), indicating that the inhibitory effects of the two domains of AnxA2 on network formation are specific.

### AnxA2 antibodies inhibit capillary-like network formation

**Figure 3:**
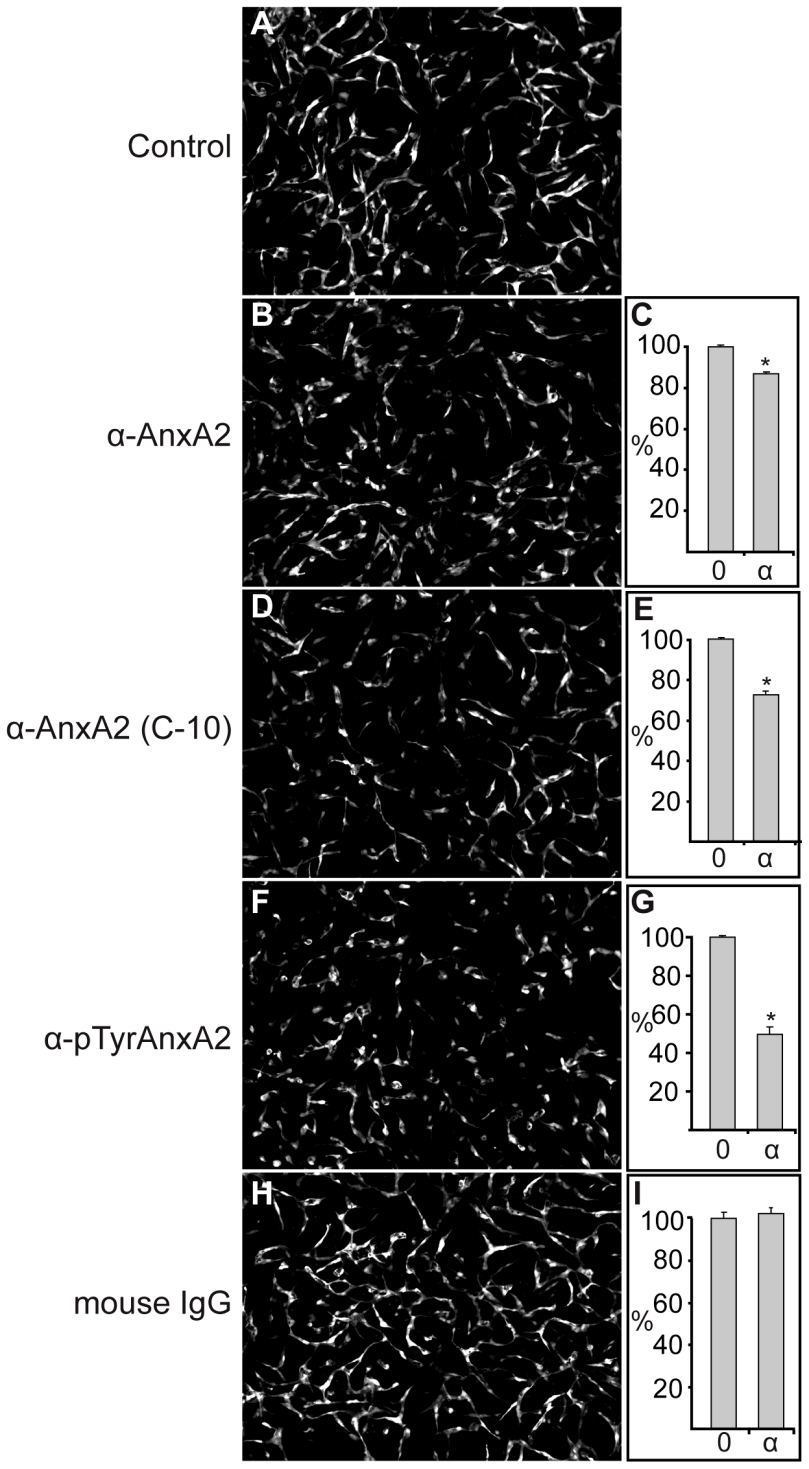
The effect of monoclonal AnxA2 antibodies on the formation of an *in vitro* capillary-like network. Co-cultures of SMCs and GFP-expressing HUVECs were untreated (**A**), or treated with 4 µg/200 µl monoclonal AnxA2 antibodies (BD Biosciences) (**B**), (C-10; Santa Cruz), (**D**), Tyr23 AnxA2 specific antibodies (Santa Cruz) (**F**) or normal mouse IgG (**H**) at 2 h after seeding. After 72 h incubation, images were taken at 10× magnification. The tube total length was analysed (**C**, **E**, **G** and **I**) and expressed as percentage relative to the corresponding untreated EC control (100%) (**A**), as described for Figure 1. Results (**C**, **E**, **G** and **I**) are the mean ± SEM of 3 independent experiments each. Statistical significance was determined by the two-tailed Student's t-test (*P<0.05).

**Figure 4:**
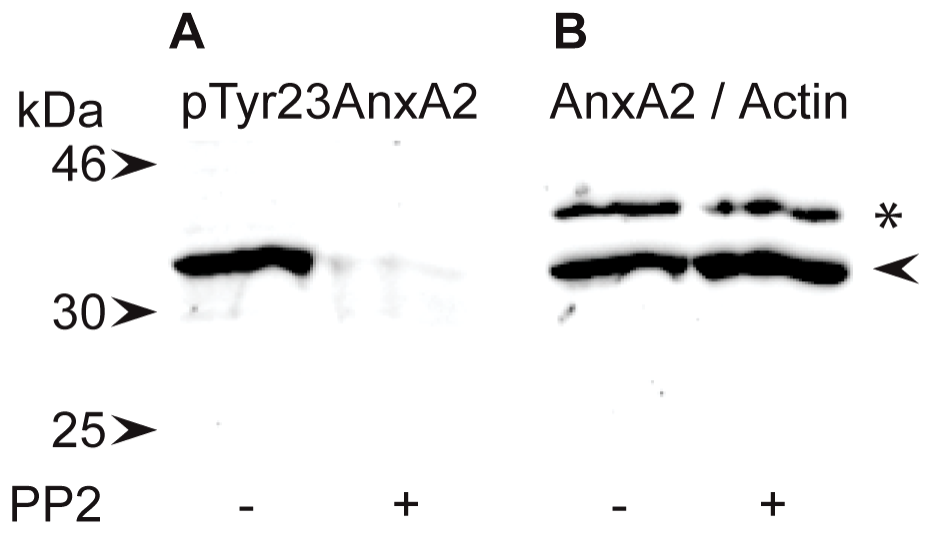
Tyr23 phosphorylated AnxA2 is present in the ECM of confluent HUVECs. HUVECs were incubated for 30 min in the absence (control) or presence of 50 µM PP2 before harvesting. The control fraction (ECM) was obtained in the presence of 200 µM ortho-vanadate to inhibit dephosphorylation. 100 µg of EGTA-released extracellular proteins were subjected to 10% SDS-PAGE and subsequently transferred to a nitrocellulose membrane for the detection of AnxA2 by Western blot analysis using monoclonal antibodies directed against pTyr23 AnxA2 (**A**) or AnxA2 (BD Biosciences) (**B**). Selected standards are indicated by arrowheads to the left. AnxA2 and actin (as a loading control) are indicated by an arrowhead and asterisk, respectively.

The direct involvement of endogenous extracellular AnxA2, present on human umbilical vein endothelial cells (HUVECs) [8], SMCs, or possibly both cell types [50], in the formation of a capillary-like network in the co-culture system is also indicated by the inhibitory effects of three different monoclonal AnxA2 antibodies on this process (Figure 3). The C-10 antibody raised against the 50 most N-terminal amino acids of AnxA2 recognises full-length protein as well as AnxA2-D_I_, while the antibody from BD Biosciences recognises amino acids 123–321 of AnxA2, but not AnxA2-D_I_ or the soluble mutated AnxA2-D_IV_ (Figure S3). The inhibitory effects of these two antibodies were not as pronounced (Figure 3, B–E) as that of the monoclonal antibody specific for the Tyr23 phosphorylated form of AnxA2 (Figure 3, F and G). In line with these results, EGTA treatment of HUVECs indicated the presence of Tyr23 phosphorylated AnxA2 at the extracellular side or the ECM of these cells (Figure 4A). The detection of AnxA2 by this antibody was abolished when Tyr phosphorylation was inhibited by using the Src kinase family inhibitor, 3-(4-chlorophenyl) 1-(1,1-dimethylethyl)-1H-pyrazolo[3,4-d]pyrimidin-4-amine (PP2; 50 µM) (Figure 4A), indicating its specificity (Figure 4, compare A and B). Furthermore, normal mouse IgG exerted no effect (Figure 3, H and I). These results further indicate the involvement of AnxA2, and in particular its Tyr23 phosphorylated form in neovascularisation.

### Domains I and IV of AnxA2 disrupt preformed capillary-like networks

**Figure 5:**
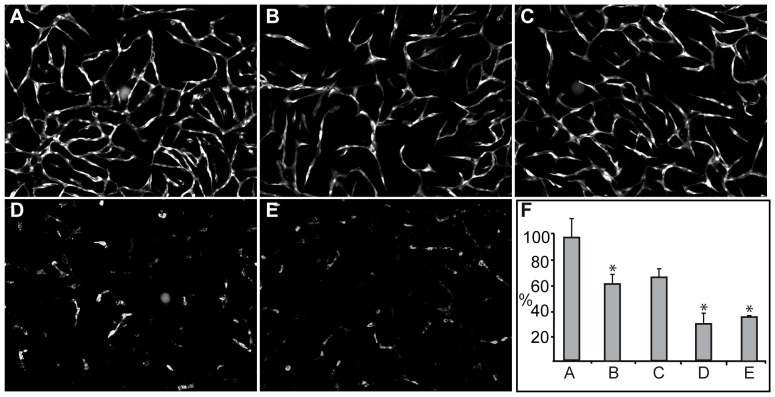
The effects of AnxA2, S100A10, AnxA2-D_I_, or AnxA2-D_IV_ on *in vitro* preformed capillary-like networks. Co-cultures with a preformed EC network were treated with 15 µM AnxA2 (**B**), S100A10 (**C**), AnxA2-D_I_ (**D**), or AnxA2-D_IV_ (**E**). After 72 h incubation, images were taken at 10× magnification. The degree of disruption of the mature vascular network was quantified (**F**) as described for Figure 1. The tube total length is expressed as percentage relative to the untreated EC control (100%) (**A**). Results (**F**) are the mean ± SEM of 3 independent experiments each. Statistical significance was determined by the two-tailed Student's t-test (*P<0.05).

Previous studies showed that the formation of capillary-like networks in the co-culture system is dependent on VEGF expressed by the SMCs [29]. In addition, network formation involves actin-dependent migration of HUVECs, which is stimulated by extracellular signals [51]. The addition of exogenous AnxA2-D_I_ or AnxA2-D_IV_ to confluent HUVEC monocultures did not appear to induce any morphological changes (data not shown), indicating that these domains may sequester factors, such as VEGF, provided by the SMCs. Once established, the endothelial networks become largely independent of VEGF [29] as the inhibitor PTK787 largely loses its ability to disrupt network formation. In particular, it does not affect the morphology of the ECs (Figure S4). Therefore, to investigate whether the observed inhibitory effects on network formation in the co-culture system are VEGF-independent, exogenous S100A10, AnxA2, AnxA2-D_I_ or AnxA2-D_IV_, were added at the highest concentration (Figure 2; 15 µM) to the preformed capillary-like networks. Both AnxA2 and S100A10 (Figure 5, B and C) were able to partially disrupt the established capillary-like networks. By contrast, AnxA2-D_I_ and AnxA2-D_IV_ destroyed the networks more or less completely and caused the HUVECs to change their morphology from an elongated to a more rounded shape (Figure 5, D and E), indicating disruption of cell-cell contacts between the ECs and rearrangement of the actin filament system.

### Domains I and IV of AnxA2 do not inhibit transmigration of HUVECs via plasmin-mediated fibrinolysis

**Figure 6:**
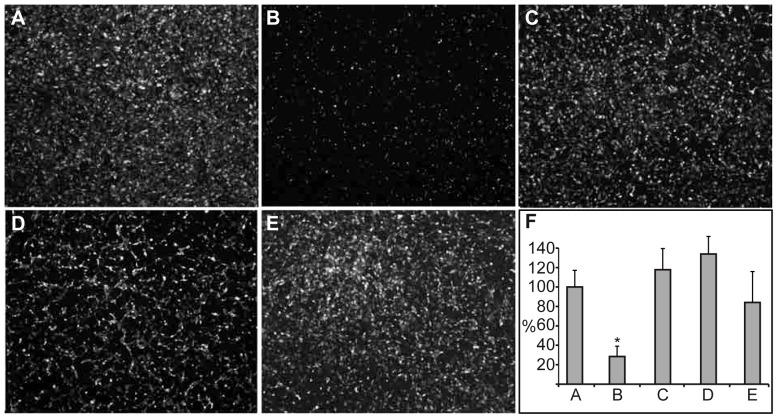
AnxA2-D_I_ and AnxA2-D_IV_ do not inhibit the migration of GFP-expressing HUVECs through a fibrin clot in a trans-well assay. Images of GFP-expressing HUVECs migrated to the backside of a filter with a fibrin clot on its upper side, onto which the HUVECs were seeded. Control HUVECs (A); HUVECs incubated with 100 nM PTK787 (B), 15 µM lysozyme (C), AnxA2-D_I_ (D), or AnxA2-D_IV_ (E), which were present in both the upper and lower chambers. Migration is expressed as percentage relative to the untreated EC control (100%) (A). Results (F) are the mean ± SEM of 3 independent experiments each. Statistical significance was determined by the two-tailed Student's t-test (*P<0.05).

Exogenously added AnxA2-D_I_ and AnxA2-D_IV_ could inhibit network formation in the co-culture system either via inactivation of the endogenous AnxA2 heterotetrameric complex in plasmin generation or reorganisation of the actin filament system due to disruption of cell-cell contacts. To distinguish between these possibilities, the ability of HUVECs to migrate in a trans-well system with or without a fibrin clot was assayed. HUVECs were capable of transmigration both in the absence and presence of AnxA2-D_I_ or AnxA2-D_IV_ (results not shown). The transmigration of HUVECs through a fibrin clot is dependent on the generation of plasmin from plasminogen due to the cleavage of cross-linked fibrin by plasmin [44]. As compared to the control assay (Figure 6A), AnxA2-D_I_ or AnxA2-D_IV_ had little or no effect on this process (Figure 6, D and E, respectively). By contrast, considerably reduced transmigration (∼70% inhibition) of HUVECs was observed in the presence of PTK787 (negative control; Figure 6B). Thus, it appears that exogenously added domains I or IV of AnxA2 do not inhibit network formation in the co-culture system by competing with endogenous AnxA2 for plasminogen/plasmin.

### Domains I and IV of AnxA2 do not bind actin

**Figure 7:**
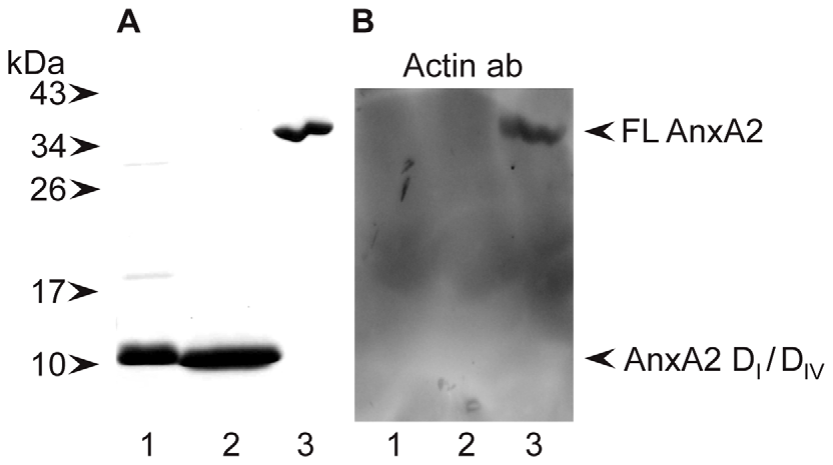
AnxA2-D_I_ and AnxA2-D_IV_ do not bind to α-actin. ∼10 µg of AnxA2-D_I_ (∼55 µM) (lane 1), AnxA2-D_IV_ (∼55 µM) (lane 2) and 5 µg of AnxA2 (∼6 µM) (lane 3) were separated by 15% SDS-PAGE (**A** and **B**) and transferred to a nitrocellulose membrane (**B**). Proteins were visualised by Coomassie Brilliant Blue staining (**A**). Far-Western (**B**); after denaturation and renaturation as described in Methods, the proteins were subjected to an actin overlay assay by incubation ON with 10 µg/ml α-actin and subsequent detection of bound actin by monoclonal actin antibodies. The positions of full-length (FL) AnxA2, and the domains I and IV of AnxA2 are indicated by arrowheads to the right. Selected standards are indicated by arrowheads to the left.

Sequence alignment of domains I and IV of AnxA2 revealed that they possess two separate regions containing partial homology (see Materials and Methods). Lys279 and Lys281 in the most N-terminal region of AnxA2-D_IV_, which are present in one of the regions of homology, have previously been shown to be involved in phosphatidylinositol 4,5-bisphosphate (PtdIns(4,5)P_2_) binding in AnxA2-D_IV_ [22]. It has been suggested that PtdIns(4,5)P_2_ is involved in the coordination of the different phases of the angiogenic programme [52]. This lipid contributes to the stimulation of actin polymerisation and is important for establishing cytoskeleton-plasma membrane linkages. AnxA2 is recruited to actin-rich membrane areas characterised also by high concentrations of cholesterol and PtdIns(4,5)P_2_ and may thus serve as a possible linker between these three ligands [16], [24]. This suggests that the two AnxA2 domains could function in the reorganisation of the actin filaments necessary for angiogenesis. Although these domains share some similarity, AnxA2-D_IV_ is able to bind mRNA [53], while AnxA2-D_I_ is not (results not shown). Recent studies have shown that the rearrangement of junctions between ECs involves cytoskeletal rearrangements [54]. To investigate whether AnxA2-D_I_ or AnxA2-D_IV_ inhibit network formation in the co-culture system and disrupt a pre-existing EC network by binding to actin, in this case, most likely extracellular actin [55], nitrocellulose blots containing AnxA2-D_I_, AnxA2-D_IV_ or full-length AnxA2 were incubated with G-actin whose binding was detected with specific monoclonal antibodies. As expected, full-length AnxA2 binds actin [16] while AnxA2-D_I_ does not (Figure 7B, lanes 1 and 3). Furthermore, AnxA2-D_IV_, which has been suggested to harbour actin-binding sites [17], does not bind actin (Figure 7B, lane 2), possibly due to the mutations introduced in this domain to make it soluble. Since AnxA2-D_IV_ is only partially folded [41], it cannot be ruled out that the secondary structure of the domain required for actin binding has been affected. However, these mutations were aimed to affect only the hydrophobic amino acid residues involved in interfacial contacts with the other domains. Moreover, it has recently been suggested that actin binds to the N-terminal tail of AnxA2 [56], which is in better agreement with our data. In conclusion, it seems unlikely that the inhibitory effects of the two domains of AnxA2 on network formation and maintenance in the co-culture system are linked to the actin-binding property of the protein.

### Domains I and IV of AnxA2 cause VE-cadherin internalisation and increase the formation of filopodia in preformed capillary-like networks

**Figure 8:**
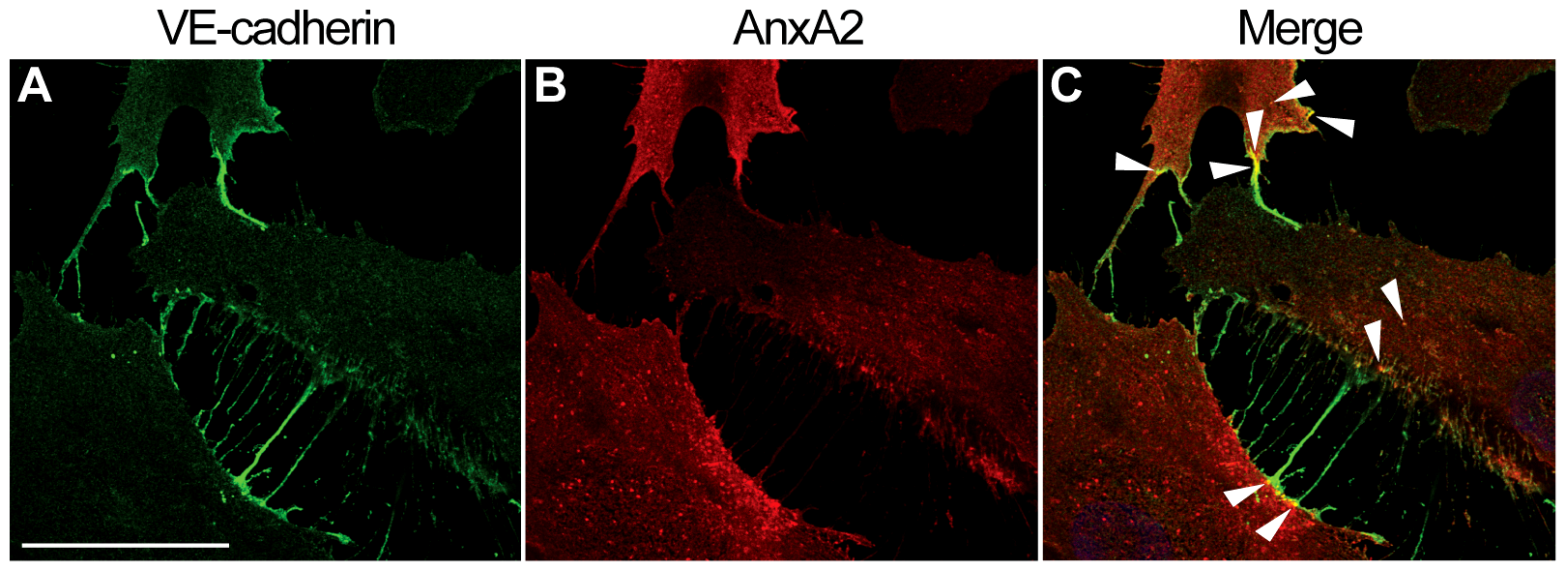
AnxA2 and VE-cadherin co-localise in endosome- and filopodia-like structures in sub-confluent HUVECs grown as a monolayer. The cells were fixed in 3% paraformaldehyde and permeabilised with 0.05% Triton X-100 in PBS before further processing for dual label immunofluorescence using antibodies directed against endogenous VE-cadherin (A) and AnxA2 (B). (C) shows the merged image. Several sites of VE-cadherin and AnxA2 co-localisation are indicated by arrowheads. Bar, 40 µm.

In HUVECs grown as monocultures, AnxA2 is present in punctate, endosome-like structures in the peripheral cytoplasm and in the vicinity of the plasma membrane. Indeed, some of these structures also contain the early endosomal antigen 1 (EEA1) (Figure S5), consistent with the presence of AnxA2 in early endosomes [57], [58], [59]. Some VE-cadherin-positive structures are also positive for EEA1 (Figure S5) in agreement with earlier findings indicating the presence of VE-cadherin in endosomes [60]. Furthermore, in HUVEC monolayers VE-cadherin and AnxA2 partially co-localise in structures (Figure 8C) similar to the AnxA2- and EEA1-positive endosomes in HeLa cells [61]. In HeLa cells, more extensive co-localisation of AnxA2 and EEA1 was observed, possibly due to the increased Tyr23 phosphorylation of AnxA2, which is a typical feature of cancer cells such as HeLa [62] and Tyr23 phosphorylation of AnxA2 is essential for its proper endosomal association [63]. VE-cadherin and AnxA2 also appear to co-localise in filopodia-like structures protruding from the surface of the sub-confluent HUVECs (Figure 8C), in accordance with the suggested role of AnxA2 in the initial stages of the formation of cell-cell junctions [64].

**Figure 9:**
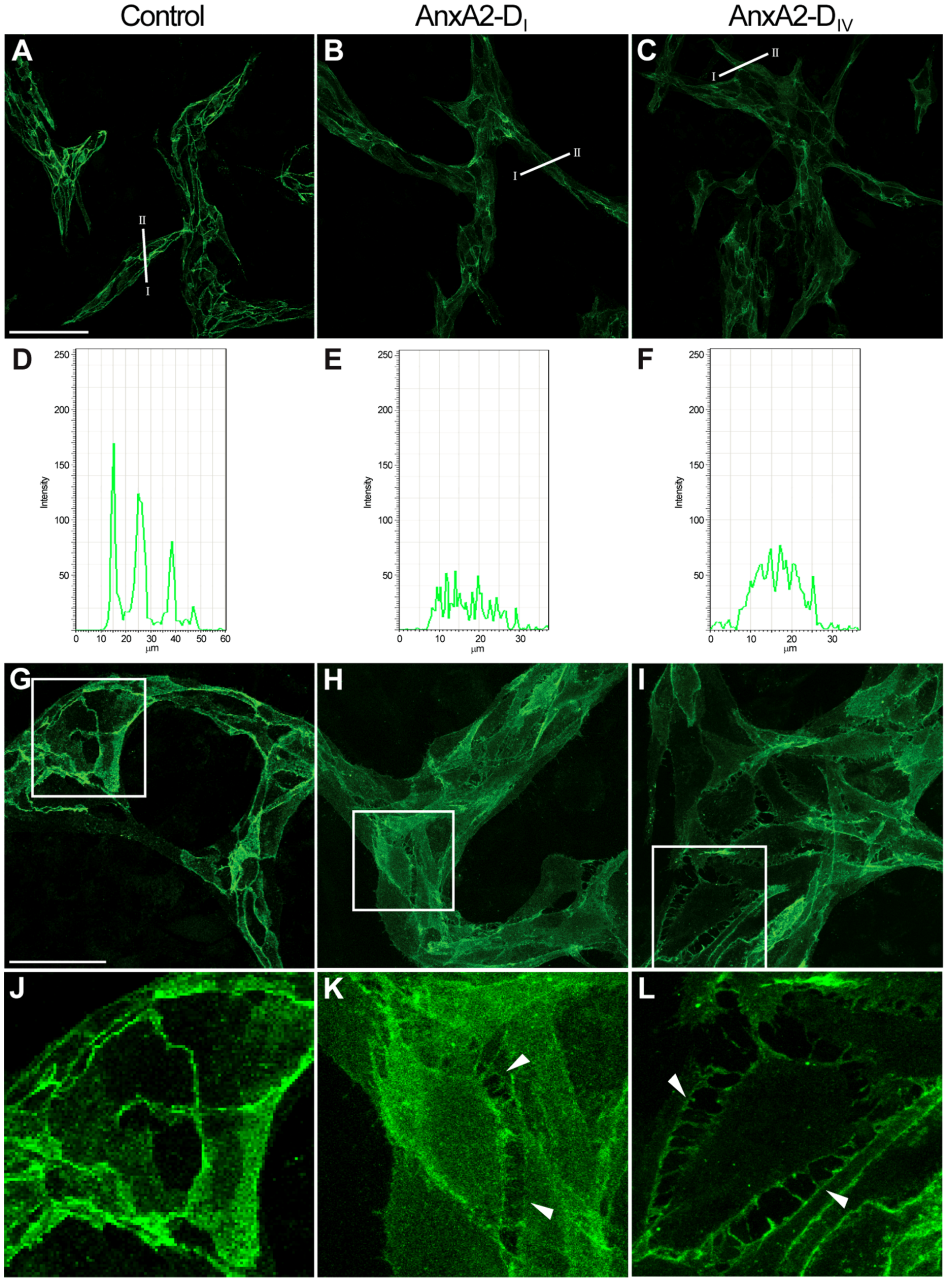
AnxA2-D_I_ and AnxA2-D_IV_ disrupt *in vitro* preformed capillary-like networks. Co-cultures with a preformed EC network were untreated (**A**, **D**, **G** and **J**), or treated for 3 h with 15 µM AnxA2-D_I_ (**B**, **E**, **H** and **K**) or AnxA2-D_IV_ (**C**, **F**, **I** and **L**). The cells were fixed in 3% paraformaldehyde and permeabilised with 0.05% Triton X-100 in PBS before processing for immunofluorescence using antibodies against endogenous AnxA2 (BD Biosciences). Note the filopodia-like structures (arrowheads) particularly in Panels **K** and **L**. Inserts in Panels **G**, **H** and **I** are magnified and shown in Panels **J**, **K** and **L**, respectively. Graphical representation (**D**–**F**) of the fluorescence intensity profiles determined for cross-sections of the cell as indicated in **A**–**C**. The orientation of the sections (from I to II) corresponds to intensity profiles from left to right in **D**, **E** and **F**. Bars, 100 µm (**A**–**C**) or 50 µm (**G**–**I**).

Confocal microscopy was also used to study the effect of AnxA2-D_I_ and AnxA2-D_IV_ on the integrity of VE-cadherin-mediated contact sites between ECs in the co-culture system. As compared to control (Figure 9, A, D, G and J), the addition of AnxA2-D_I_ (Figure 9, B, E, H and K) or AnxA2-D_IV_ (Figure 9, C, F, I and L) to the preformed mature EC network reduced the strong cell surface signal of VE-cadherin at sites where adjacent cells make intimate contact. Simultaneous increase in the cytoplasmic signal indicates that the presence of the AnxA2 domains in the medium promotes internalisation of VE-cadherin. Furthermore, ECs, in particular cells at network branch points, detach from each other and evidently become mobile after the addition of AnxA2-D_I_ (Figure 9, H and K) or AnxA2-D_IV_ (Figure 9, I and L), as indicated by the formation of the filopodia-like structures. Reliable comparison of the localisation of VE-cadherin and AnxA2 in HUVECs in the co-culture system was not possible due to the intensive AnxA2 signal of the confluent layer of SMCs beneath the HUVECs (results not shown). Interestingly, it has been shown that VE-cadherin antibodies, like domains I and IV of AnxA2, inhibit the formation of capillaries, as well as disrupt preformed capillaries [65]. In conclusion, these results suggest that the AnxA2-D_I_ and AnxA2-D_IV_ disrupt the capillary-like network by affecting the endothelial cell-cell contacts by competing with endogenous AnxA2 for interaction with VE-cadherin.

## Discussion

By binding simultaneously to tPA and plasminogen, AnxA2 increases the activation of plasmin by about 60-fold [66]. tPA binds to amino acids 7–12 in the AnxA2 N-terminus in which Cys8 is crucial for the interaction [67], [68]. However, it has been proposed that in addition other regions of AnxA2 could also be involved in the binding of the protein to tPA [47]. It has been suggested that plasminogen is activated after binding to Lys307 in AnxA2, which becomes exposed after protease cleavage [15]. AnxA2 in its heterotetrameric form significantly inhibited network formation in the co-culture system whereas the two subunits alone exerted negligible anti-angiogenic effects (Figure 1). One possibility is that S100A10 is the active protein in the heterotetrameric complex. Alternatively, the binding of S100A10 results in the unmasking of a site in AnxA2 which is important for the binding of extracellular ligands. The latter explanation appears more plausible since a soluble s (Δ20AnxA2; starting at Pro21, the first visible amino acid in the full-length AnxA2 crystal structure) inhibited network formation by ∼50% (Figure S6).

To identify regions of AnxA2 involved in angiogenesis, two soluble domains of the protein were tested as potential inhibitors of this process in the co-culture system. AnxA2-D_IV_ is postulated to harbour the plasminogen-binding site [15], as well as the actin-binding site(s) [17], while AnxA2-D_I_, in addition to the N-terminus, could provide sites for binding to tPA [47]. Both domains of AnxA2 were potent inhibitors of VEGF-dependent network formation in the co-culture system. Interestingly, AnxA2 expression is regulated and increased in a mouse model of ischemic retinopathy via a VEGF/VEGF receptor 2/PKCβ pathway [38]. In addition, VEGF induces Tyr phosphorylation and nuclear translocation of Activator of Transcription 6 (STAT6) [69], which interacts with AnxA2 in prostate cancer cells [70], suggesting that one of the angiogenic effects of VEGF is to regulate the expression of AnxA2 directly or indirectly.

The VEGF-dependent stages of angiogenesis involve the activation of tPA [68]. By contrast, the preformed capillary-like network in the co-culture system is largely VEGF-independent and its integrity relies mainly on ECM deposition and homo- and heterotypic cell-cell contacts between ECs and SMCs [29]. It has been shown that AnxA2 is present in the extracellular surface of HUVECs (Figure 4) [15]. Exogenously added domains I or IV of AnxA2 could therefore prevent vascularisation in the co-culture by two different mechanisms; i.e. either by competing with endogenous AnxA2 for plasminogen/plasmin/tPA and/or other factors involved in the stabilisation of cell-cell contact sites between HUVECs. To investigate whether the inhibitory effect of exogenously added AnxA2 is solely VEGF-dependent, the two domains of AnxA2 were added to the co-culture system after the network formation was completed. Exogenously added AnxA2-D_I_ and AnxA2-D_IV_ efficiently disrupted the preformed mature endothelial network formed by the HUVECs in the co-culture (Figure 5). Full-length AnxA2 and S100A10 were also able to disrupt the preformed network, although less effectively, indicating that AnxA2 participates at different VEGF-dependent and independent stages of angiogenesis.

Studies of pancreatic cells have shown that the sorting of AnxA2 to the cell surface is Tyr23 phosphorylation-dependent, contributes to epithelial-to-mesenchymal transition and is required for tumour invasion and metastasis [62]; i.e. processes that all involve reorganisation of the actin filament system. In agreement with this, Tyr23 phosphorylation of AnxA2 has been shown to be important in actin reorganisation, cell scattering and branching morphogenesis [6], [71], [72], most likely by regulating cofilin phosphorylation [71]. Actin reorganisation in response to extracellular stimuli, mainly through receptor Tyr kinases, results in altered cell motility. The Tyr23 phosphorylated AnxA2 is also present on the extracellular cell surface (Figure 4). The inhibitory effects of monoclonal AnxA2 antibodies on the formation of the capillary-like network also indicate a crucial role of extracellular AnxA2, in particular the Tyr23 phosphorylated form, in neovascularisation (Figure 3). In line with these data, it was recently shown that AnxA2 antibodies inhibit neoangiogenesis and human breast tumour growth in a xenograft model [73]. Furthermore, AnxA2 antibodies inhibit pancreatic ductal adenocarcinoma metastases, thus prolonging survival [62]. This finding is corroborated by another study showing that treatment of the highly invasive/metastatic MDA-MB231 cell line with AnxA2 antibodies leads to decreased cell migration [74].

Exogenously added AnxA2-D_IV_ only disrupts cell-cell contacts between HUVECs in the network when they are grown in the presence of SMC, but has no effect on confluent HUVEC monolayers grown in the presence of VEGF (data not shown). Thus, cell-cell contacts between SMC and HUVEC are important for the AnxA2-mediated effect. AnxA2 has been reported to contain an actin-binding site in its domain IV [17], but the effects on the co-culture system exerted by AnxA2-D_I_ and AnxA2-D_IV_ are not mediated through their direct binding to actin (Figure 7). However, since the HUVECs in the disintegrated network change morphology from elongated to more rounded, it is clear that rearrangements of the cytoskeleton are involved. In this connection, it should be noted that AnxA2 binds VE-cadherin in the adherens junctions of ECs [18], AHNAK in tight junctions in kidney cells [75], [76], and the carcinoembryonic antigen cell adhesion molecule-1 (CEACAM1), a potent VEGF-mediated pro-angiogenic factor in cell-cell adhesions in several cell types [77]. These findings are consistent with the involvement of AnxA2 in cell-cell contacts and dynamic membrane-microfilament interactions. These interactions could be regulated by specific signalling pathways via ligand binding to extracellular AnxA2. It has been shown that sphingosine 1-phosphate mediates the translocation of AnxA2 from the cytosol to the plasma membrane where it subsequently complexes with VE-cadherin and controls AKT activation [78]. The VE-cadherin-based complex consists of α-, β- and γ-catenins, actin and AnxA2 which docks the complex to lipid rafts [18]. VE-cadherin is essential for the maintenance and control of adhesion between ECs in vascular tubes [79]. The interaction of AnxA2 with the VE-cadherin-based complex is very labile and has been proposed to act as a switch between an angiogenic and a quiescent state [18]. VEGF stimulation of ECs causes internalisation of VE-cadherin via clathrin-mediated endocytosis [80] and extracellular AnxA2 is also internalised via the same mechanism [81]. AnxA2 has been reported to regulate endothelial morphogenesis by binding to VE-cadherin after sphingosine 1-phosphate stimulation and thus stabilising adherens junctions [78]. The partial co-localisation of AnxA2 and VE-cadherin with each other and with EEA1 (Figures 8 and S5) in endosome-like structures in HUVEC monolayers indicates that the inhibitory effects of the domains I and IV of AnxA2 on network formation do not involve the internalisation of AnxA2 although cellular uptake of AnxA2 may increase in the co-cultures when treated with domains I and IV. However, when MBP-AnxA2-D_IV_ was added to the co-culture system, it was detected by MBP antibodies only in the medium, and not in the EGTA-released protein fraction nor in the cell lysate (results not shown), arguing against the latter possibility and consistent with the idea that inhibition of network formation by the domains is mainly mediated by sequestration of soluble extracellular factors.

In conclusion, we have shown that the exogenously added domains I and IV of AnxA2 are potent inhibitors of VEGF-dependent network formation in the co-culture system. Furthermore, the domains are also potent in disrupting the preformed capillary-like networks that are VEGF-independent. The effects are most likely mediated by their competition with endogenous extracellular AnxA2 for factors involved in cell-cell adhesion, whereas internalisation of AnxA2 is not likely to be part of the inhibitory mechanism. It is possible that in addition to Tyr23 phosphorylation, the binding of AnxA2 to S100A10 is required for the translocation of AnxA2 to the plasma membrane. This in turn may expose site(s) important for binding to specific factors that are involved in vascularisation. In line with this, truncation of the first 20 amino acids of AnxA2 increases the anti-angiogenic effect of exogenously added AnxA2 in the co-culture system (Figure S6).

## Supporting Information

Figure S1 **Purified S100A10.** 5 µg of purified S100A10 was separated by 12% SDS-PAGE and visualised by Coomassie Brilliant Blue staining. Selected standards are indicated by arrowheads to the left.
(TIF)

Figure S2 **Circular dichroism measurements of AnxA2-D_I_ in the far-UV region.** (**A**) The far-UV CD spectrum was recorded for 40 µM AnxA2-D_I_ at pH 8 (20 mM Tris) at 20°C and was background corrected. The observed optical activity is expressed as the mean residue molar ellipticity [θ]_MRW_ (deg cm^2^ dmol^−1^). (**B**) CD-monitored thermal disruption (range 20–90°C) of α-helicity of 40 µM AnxA2-D_I_. The change in ellipticity at 222 nm was measured at pH 8 at a heating rate of 40°C/h. The observed optical activity is expressed as the mean residue molar ellipticity [θ]_MRW_ (deg cm^2^ dmol^−1^). The apparent transition temperature (Tm) was determined from the first derivative of the curve.
(TIF)

Figure S3 **The recognition of the AnxA2-D_I_ and AnxA2-D_IV_ by monoclonal AnxA2 antibodies.** 2 µg of AnxA2 (lanes 1 and 4), AnxA2-D_I_ (lanes 2 and 5) and AnxA2-D_IV_ (lanes 3 and 6) were subjected to 15% SDS-PAGE and Western blot analysis using monoclonal antibodies against AnxA2 from BD Biosciences (**A**) or Santa Cruz (C-10) (**B**). The positions of full-length (FL) AnxA2, AnxA2-D_I_ and AnxA2-D_IV_ are indicated by arrowheads to the right.
(TIF)

Figure S4 **The effect of PTK787 on preformed capillary-like networks.** Co-cultures with a preformed EC network were treated with 100 nM PTK787 (**B**). After 72 h incubation, images were taken at 10× magnification. It should be noted that the ECs in (**B**) were less dense than in control (**A**) when treatment with PTK787 started.
(TIF)

Figure S5 **AnxA2 and VE-cadherin partially co-localise with EEA1 in endosome-like structures in sub-confluent HUVECs grown as monolayer.** The cells were fixed in 3% paraformaldehyde and permeabilised with 0.05% Triton X-100 in PBS before further processing for dual label immunofluorescence using antibodies directed against endogenous AnxA2 (**A**), VE-cadherin (**D**) and EEA1 (**B** and **E**). (**C** and **F**) show the corresponding merged images. Several sites where AnxA2 or VE-cadherin co-localise with EEA1 are indicated by arrowheads (C and F; inserts). Bar, 20 µm.
(TIF)

Figure S6 **The effect of Δ20AnxA2 on the formation of an** ***in vitro*** **capillary-like network.** Co-cultures of SMCs and GFP-expressing HUVECs were untreated (**A**) or treated with 15 µM Δ20AnxA2 (AnxA2 lacking the first 20 N-terminal amino acids) (**B**) at 2 h after seeding. The images were taken at 10× magnitude after 72 h incubation. The tube total length (**C**) is expressed as percentage relative to the untreated EC control (100%) (**A**) using the Attovision and BD Image Data Explorer programmes. Results (**C**) are the mean (± SEM) of 3 independent experiments each. Statistical significance was determined by the two-tailed Student's t-test (*P<0.05).
(TIF)
