# Correction: Domains I and IV of Annexin A2 Affect the Formation and Integrity of *In Vitro* Capillary-Like Networks

**DOI:** 10.1371/annotation/c39f6c86-6829-4bd5-9841-19b8882ea421

**Published:** 2013-10-16

**Authors:** Aase M. Raddum, Lasse Evensen, Hanne Hollås, Ann Kari Grindheim, James B. Lorens, Anni Vedeler

There was an error in the second sentence of the legend of Figure 3. The correct sentence is: “The cells were fixed in 3% paraformaldehyde and permeabilised with 0.05% Triton X-100 in PBS before processing for immunofluorescence using antibodies against endogenous VE-cadherin.”

