# Supplementary figures and images for: Domains I and IV of Annexin A2 Affect the Formation and Integrity of In Vitro Capillary-Like Networks
Source: PLoS One. 2013 Mar 29;8(3):e60281. doi: 10.1371/journal.pone.0060281 (PMC3612057; doi:10.1371/journal.pone.0060281)

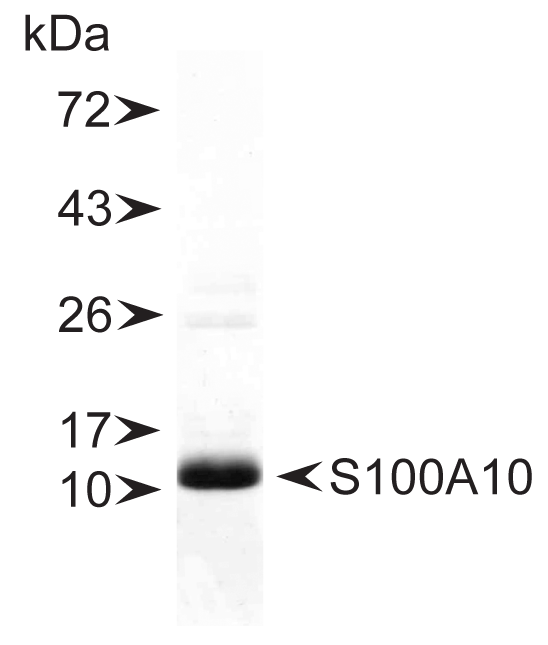

Supplement: Figure S1 — Purified S100A10. 5 µg of purified S100A10 was separated by 12% SDS-PAGE and visualised by Coomassie Brilliant Blue staining. Selected standards are indicated by arrowheads to the left. (TIF) [file pone.0060281.s001.tif]

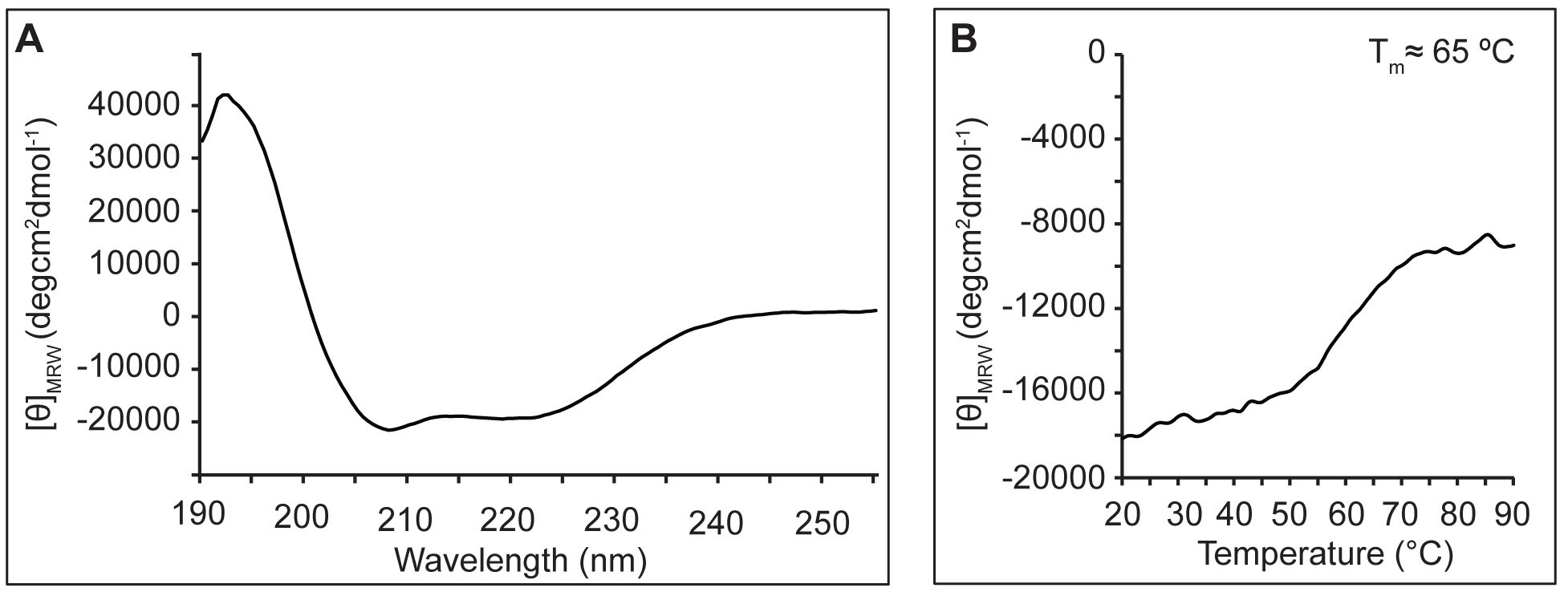

Supplement: Figure S2 — Circular dichroism measurements of AnxA2-DI in the far-UV region. (A) The far-UV CD spectrum was recorded for 40 µM AnxA2-DI at pH 8 (20 mM Tris) at 20°C and was background corrected. The observed optical activity is expressed as the mean residue molar ellipticity [θ]MRW (deg cm2 dmol−1). (B) CD-monitored thermal disruption (range 20–90°C) of α-helicity of 40 µM AnxA2-DI. The change in ellipticity at 222 nm was measured at pH 8 at a heating rate of 40°C/h. The observed optical activity is expressed as the mean residue molar ellipticity [θ]MRW (deg cm2 dmol−1). The apparent transition temperature (Tm) was determined from the first derivative of the curve. (TIF) [file pone.0060281.s002.tif]

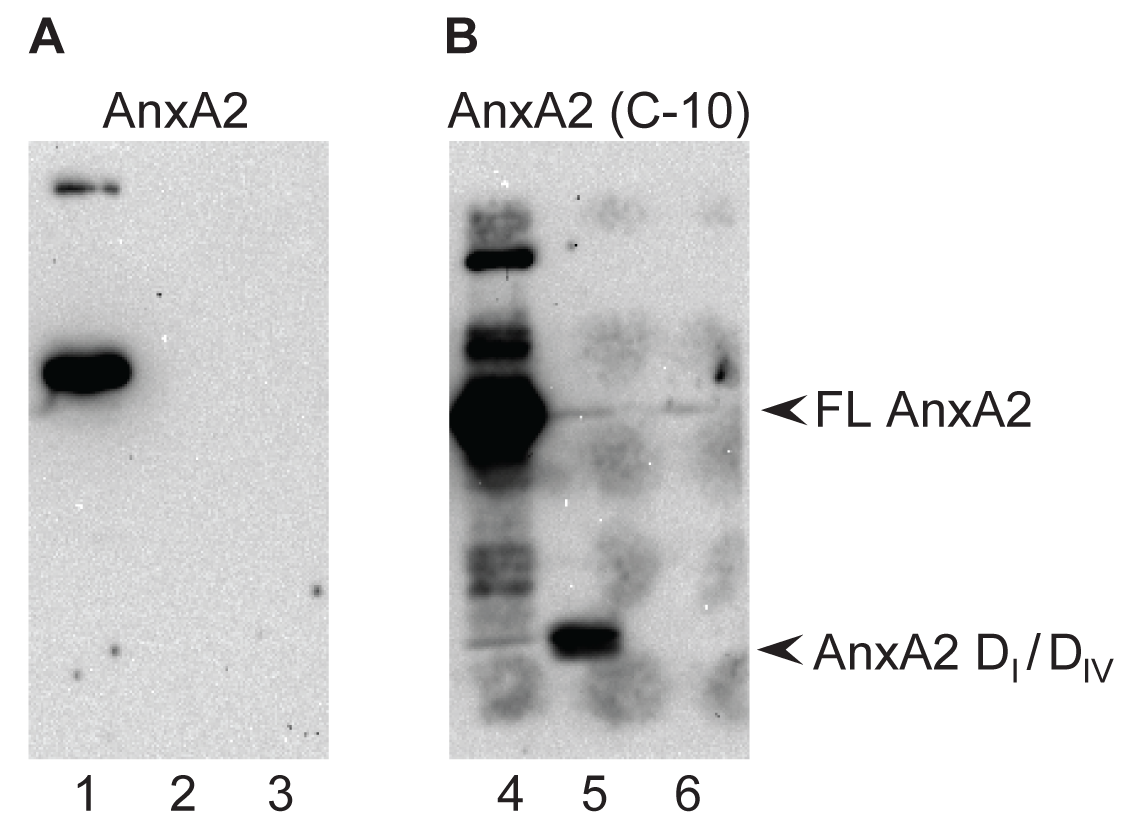

Supplement: Figure S3 — The recognition of the AnxA2-DI and AnxA2-DIV by monoclonal AnxA2 antibodies. 2 µg of AnxA2 (lanes 1 and 4), AnxA2-DI (lanes 2 and 5) and AnxA2-DIV (lanes 3 and 6) were subjected to 15% SDS-PAGE and Western blot analysis using monoclonal antibodies against AnxA2 from BD Biosciences (A) or Santa Cruz (C-10) (B). The positions of full-length (FL) AnxA2, AnxA2-DI and AnxA2-DIV are indicated by arrowheads to the right. (TIF) [file pone.0060281.s003.tif]

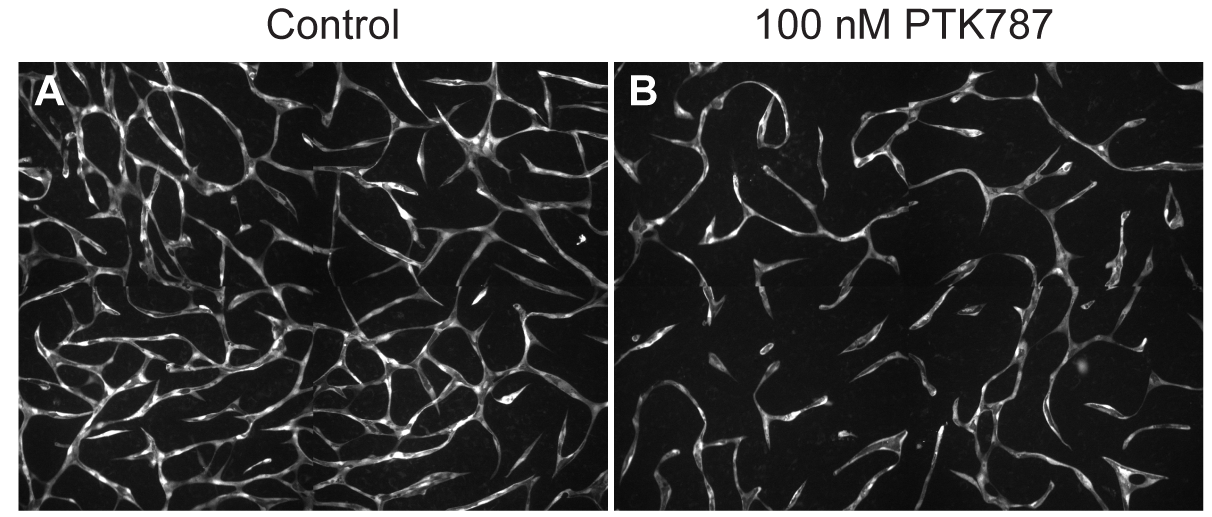

Supplement: Figure S4 — The effect of PTK787 on preformed capillary-like networks. Co-cultures with a preformed EC network were treated with 100 nM PTK787 (B). After 72 h incubation, images were taken at 10× magnification. It should be noted that the ECs in (B) were less dense than in control (A) when treatment with PTK787 started. (TIF) [file pone.0060281.s004.tif]

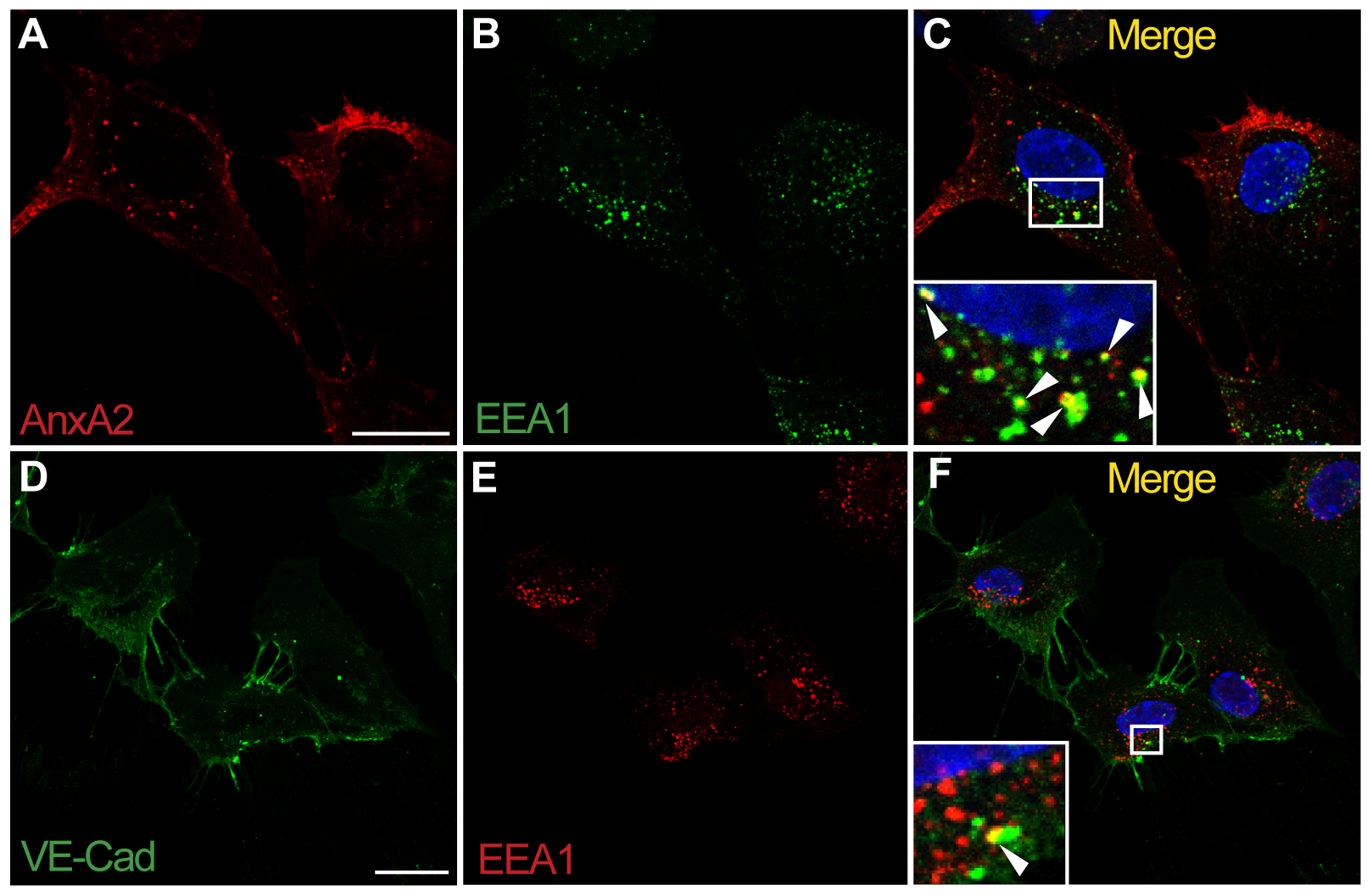

Supplement: Figure S5 — AnxA2 and VE-cadherin partially co-localise with EEA1 in endosome-like structures in sub-confluent HUVECs grown as monolayer. The cells were fixed in 3% paraformaldehyde and permeabilised with 0.05% Triton X-100 in PBS before further processing for dual label immunofluorescence using antibodies directed against endogenous AnxA2 (A), VE-cadherin (D) and EEA1 (B and E). (C and F) show the corresponding merged images. Several sites where AnxA2 or VE-cadherin co-localise with EEA1 are indicated by arrowheads (C and F; inserts). Bar, 20 µm. (TIF) [file pone.0060281.s005.tif]

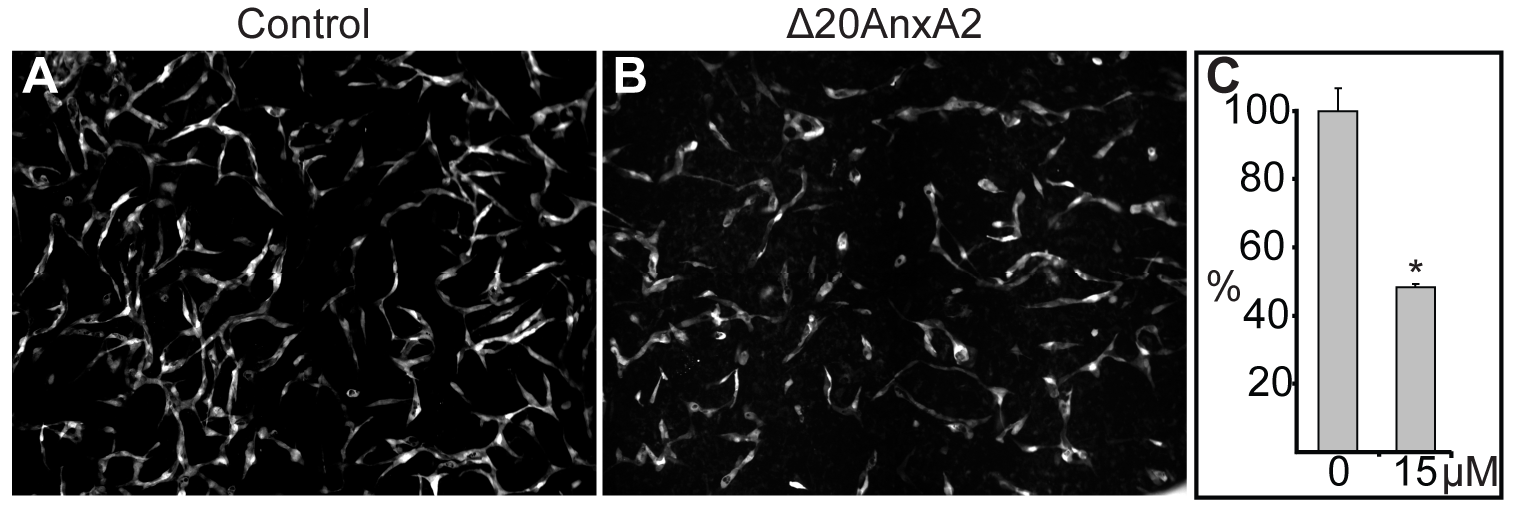

Supplement: Figure S6 — The effect of Δ20AnxA2 on the formation of an in vitro capillary-like network. Co-cultures of SMCs and GFP-expressing HUVECs were untreated (A) or treated with 15 µM Δ20AnxA2 (AnxA2 lacking the first 20 N-terminal amino acids) (B) at 2 h after seeding. The images were taken at 10× magnitude after 72 h incubation. The tube total length (C) is expressed as percentage relative to the untreated EC control (100%) (A) using the Attovision and BD Image Data Explorer programmes. Results (C) are the mean (± SEM) of 3 independent experiments each. Statistical significance was determined by the two-tailed Student's t-test (*P<0.05). (TIF) [file pone.0060281.s006.tif]
